# The avian respiratory microenvironment-immune nexus: A three-dimensional regulation model from local homeostasis to systemic defense

**DOI:** 10.1016/j.psj.2026.107518

**Published:** 2026-07-31

**Authors:** Miao Yin, Xuefeng Qi, Qingrong Jiang, Lizhen Wang, Qian Wu, Yu Xia, Xin Zhao, Miaoxi Deng, Xuemei Ding, Jinglong Yang, Xiwen Chen

**Affiliations:** a Northwest A&F University, Yangling, Shaanxi, 712100, China; b Mianyang Normal University, Mianyang, Sichuan, 621000, China; c Engineering Research Center for Monitoring and Control of Major Swine Diseases, Sichuan Provincial Department of Education, Mianyang, Sichuan, 621000, China; d Sichuan Sundaily Ecological Food Co., Ltd., Mianyang, Sichuan, 621000, China; e Sichuan Agricultural University, Chengdu, Sichuan, 611130, China; f Chongqing Academy of Animal Sciences, Chongqing, China

**Keywords:** Avian mucosal immunity, Respiratory microbiota, Gut–lung axis, Tissue-resident memory T cells, Ecological resilience

## Abstract

The avian respiratory tract is a major portal of entry for respiratory pathogens, and the dynamic interactions between the mucosal immune system and resident microbial communities constitute an important component of local defense. Although these interactions are increasingly recognized, substantial knowledge gaps remain regarding the molecular mediators of the gut–lung axis, the interplay between the microbiota and tissue-resident memory T (TRM) cells, and the mechanisms that maintain local homeostasis. This review synthesizes multidisciplinary advances in avian respiratory immunology and microbiome research. We distinguish direct evidence obtained in avian species from mechanisms inferred from mammalian studies and identify the latter as hypotheses requiring validation in birds. We discuss a putative regulatory axis linking dominant respiratory and intestinal microbial taxa, microbial metabolites such as short-chain fatty acids (SCFAs) and pyrroloquinoline quinone (PQQ), and mucosal immune effectors including secretory immunoglobulin A (sIgA) and interferon lambda 3 (IFN-λ3). We further examine the proposed pathways through which dysbiosis may contribute to the progression from local barrier disruption to systemic disease. Considering avian anatomical and immunological characteristics, including nasal-associated lymphoid tissue (NALT) and the absence of conventional draining lymph nodes in chickens and turkeys, we propose a host–microbiota–environment framework for organizing the determinants of respiratory homeostasis and disease susceptibility. Furthermore, we review the application of high-throughput sequencing and gene-editing technologies to target discovery and discuss the potential translation of microecological interventions into poultry production. Collectively, this review provides a conceptual basis for investigating avian respiratory microecology and supports the evaluation of microbiota-directed approaches as potential complements to vaccination, biosecurity, environmental management, and antimicrobial stewardship.

## Introduction

The avian respiratory tract, an open physiological barrier, serves as the primary portal of invasion for pathogens such as avian influenza virus (AIV) and infectious bronchitis virus (IBV). The avian immune system differs fundamentally from its mammalian counterpart in both lymphoid architecture and effector mechanisms (Davison et al., 2022). Its mucosal immune system must not only mount rapid defensive responses to clear pathogens but also maintain a dynamic balance between high-efficacy response and avoidance of immunopathology. However, conventional inactivated vaccines administered by injection—the predominant vaccination strategy in commercial poultry production—primarily induce systemic humoral immunity and often fail to elicit robust mucosal secretory IgA (sIgA) responses or TRM at the respiratory epithelium, thus incompletely blocking pathogen entry at the mucosal portal (Hu et al., 2025; Raha et al., 2025). It should be noted that this limitation pertains specifically to conventional injectable inactivated vaccines; emerging mucosal vector vaccines, discussed in later sections, operate through fundamentally different immunological mechanisms that can potentially address this gap. This limitation highlights the need to re-evaluate defensive mechanisms from a complementary perspective: the microecology-immune interplay. Recent integration of immunology and microbiome research has revealed that microecological homeostasis is a crucial regulatory rheostat determining both local immune efficacy and systemic disease resistance.

The respiratory microbiota is not merely a component of the physical barrier but also an essential modulator of the host immune system (Kulshreshtha et al., 2022). Studies indicate that the collapse of this homeostasis is a central driver of the cascading pathology in respiratory diseases. For instance, AIV or IBV infection can deplete core bacterial populations (e.g., *Lactobacillus species*), triggering a pro-inflammatory cytokine storm and exacerbating airway tissue damage (Luczo, 2023; Ashique, 2022). More critically, cross-organ communication mediated by the gut-lung axis reveals deeper mechanisms of systemic defense. In mammalian models, intestinal fungal dysbiosis has been associated with altered bacterial translocation and pulmonary inflammation (Yang, 2025; Ni, 2023; Thompson, 2025). Whether an equivalent fungal–bacterial translocation pathway occurs in poultry remains to be determined. Evidence from mammalian respiratory-infection models (e.g., SARS-CoV-2) suggests that disruption of the mucosal barrier can facilitate systemic dissemination of pathogens to organs such as the liver and kidneys; whether comparable pathways operate to the same extent in the avian respiratory tract requires direct investigation (Thompson, 2025; Agrawal, 2026).

Although the central role of microecology-immune interaction is widely accepted, its translation into precise prevention and control strategies faces three major mechanistic bottlenecks. First, molecular targets remain unclear: the specific effector molecules (e.g., *particular metabolites*) and downstream signaling pathways through which dominant microbiota (e.g., Lactobacillaceae) regulate the mucosal barrier are not well defined. Second, there is a lack of identified communication mediators: the material basis (metabolic/cellular mediators) for cross-boundary regulation via the gut-lung axis lacks direct evidence. Finally, there is a gap in understanding TRM interactions. General studies of TRM-cell biology have established their importance in rapid local recall responses; however, the differentiation, phenotype, and maintenance of respiratory TRM cells remain less well characterized in avian species (Liu, 2018; Xu, 2023). Furthermore, while mammalian studies suggest that microbial and metabolic signals may influence tissue-resident T-cell differentiation, direct evidence for such regulation in the avian respiratory tract remains limited. Whether the differentiation and maintenance of avian TRM depend on specific microbial-metabolic signals therefore remains unknown. To address these gaps, this review follows a logical framework of structural basis– interaction mechanisms-translational applications. We systematically outline the functional networks of avian-specific immune structures (e.g., NALT, Harderian gland), provide an in-depth explanation of the dynamic interplay between microbial homeostasis and host immunity, and construct a three-dimensional regulation model. Our aim is to fill the aforementioned knowledge gaps and provide a theoretical foundation for developing next-generation mucosal vaccines and precision intervention strategies targeting the microecology.

## Co-adaptation of avian respiratory structure and microbiota

### Immune compensatory mechanisms under anatomical constraints

Unlike mammals, chickens and turkeys lack conventional encapsulated lymph nodes. This anatomical feature has forced the evolution of unique local compensatory mechanisms within the respiratory mucosal immune system (Smialek et al., 2011; de Geus, 2015). The NALT and the Harderian gland serve as core compensatory structures, undertaking the primary tasks of antigen sampling and immune initiation. NALT, located in the lamina propria of the nasal mucosa, functions as a major inductive site rich in T/B lymphocytes and antigen-presenting cells. It can rapidly recognize and respond to pathogens such as AIV and contributes to the induction of local sIgA responses following respiratory antigen exposure (Watanabe, 2020). The Harderian gland, a unique eye-nasal-associated effector organ, not only strengthens the physical barrier through lysozyme secretion (Smialek et al., 2011) but also serves as a major residence for plasma cells, continuously supplying sIgA to the respiratory mucosa (von La Roche et al., 2026; Baba et al., 1990), thereby functionally compensating for the absence of lymph node germinal centers in birds (de Geus et al., 2012). This architecture, heavily reliant on local tissues, renders avian respiratory immunity exquisitely sensitive to changes in microenvironmental homeostasis. Precisely this lack of a structural immune filtering mechanism compels the avian respiratory tract to increases reliance on local defense mechanisms, granting the commensal microbiota colonizing this site a strategic importance not seen to the same degree in mammals.

### Spatial mapping and barrier function of the core microbiota

On this specialized anatomical interface, host and microbiota form a tightly organized spatial symbiosis map. The healthy avian respiratory tract exhibits a community structure dominated by Firmicutes and Proteobacteria, with Lactobacillaceae as a core dominant reported to be prevalent on the surfaces of nasal turbinates and tracheal mucosa (Oladokun, 2025; Bhowmick, 2025). These dominant populations are not randomly distributed but specifically occupy niches within the mucus layer, forming a biological barrier through physical occupation and competitive exclusion. This directly limits contact between conditional pathogens like *E. coli* and the NALT epithelium. Research confirms a positive correlation between the integrity of this microecological barrier and host antiviral capacity: AIV infection has been associated with substantial changes in respiratory microbial community composition, including reductions in several commensal taxa.

### Molecular basis of immune-microbial symbiotic dialogue

The host immune system and commensal microbiota engage in a finely tuned recognition-tolerance dialogue which is fundamental for maintaining homeostasis at open mucosal surfaces. Avian respiratory epithelial cells actively select for colonizing microbiota by secreting specific effector molecules. For example, the Ovocalyxin-32 protein found in eggshells and mucosal secretions has demonstrated antimicrobial and bacterial-binding properties in eggshell-associated contexts (Kulshreshtha, 2022). Whether it exerts comparable selective effects on microbial colonization in the avian respiratory tract has not been established. This selective filtering mechanism, combined with the hypo-responsiveness of innate immune receptors, Toll-like receptors (TLRs) to commensal signals, ensures that NALT remains tolerant to commensals while retaining rapid activation capacity against true pathogens (Saint-Martin, 2024). This "immune-microbial" co-evolutionary network, based on anatomical adaptation and molecular selection, constitutes the material foundation of avian respiratory defense.

## Gut-lung axis-mediated remote immune programming

The aforementioned mechanisms ensure stability at the local immune-ecological interface of the respiratory tract. However, the avian defense network extends further. A cross-organ communication system termed the gut-lung axis can remotely project immune training experiences from the gut to the respiratory tract, enabling preemptive and synergistic systemic defense.

### Remote regulation by cecal-derived metabolites

**Fig. 1:**
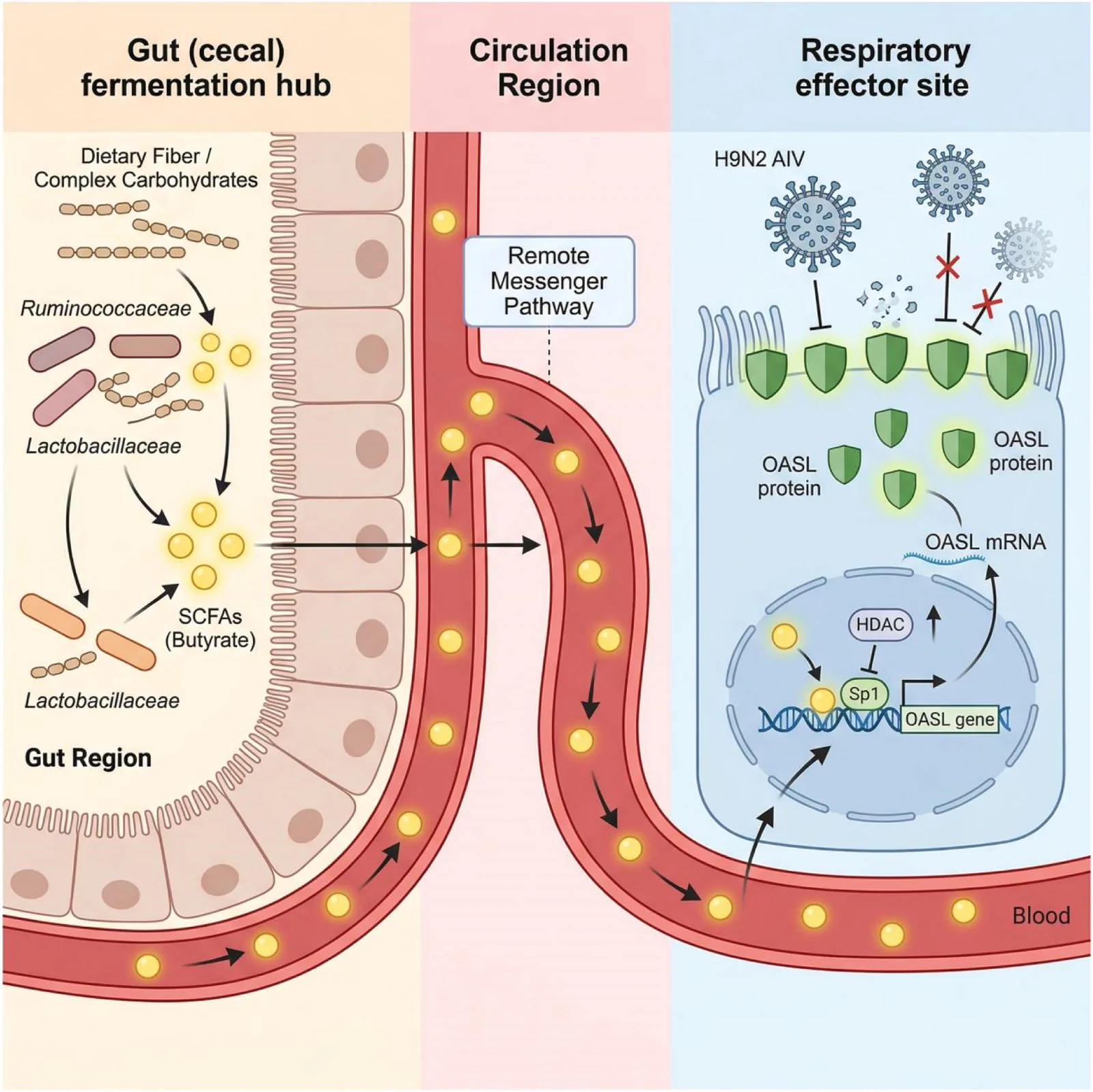
The Molecular Telegram: Microbiota-Derived SCFAs Orchestrate Respiratory Antiviral Immunity via the Sp1-OASL Axis. Description: This schematic illustrates the remote immune programming of the respiratory tract by gut-derived metabolites. Left & Center: Complex carbohydrates are fermented by cecal microbiota (e.g., Ruminococcaceae, Lactobacillaceae) into short-chain fatty acids (SCFAs), which enter the systemic circulation as "remote messengers". Right: Upon reaching the respiratory mucosa, SCFAs (acting as HDAC inhibitors) upregulate the transcription factor Sp1 in epithelial cells. This promotes the expression of the antiviral protein OASL, which directly inhibits the replication of H9N2 avian influenza virus, demonstrating a direct functional link between gut metabolism and lung innate immunity.

The well-developed avian ceca are not only primary sites for fiber fermentation but also core primary fermentation sites for immunomodulatory molecules like short-chain fatty acids (SCFAs). Under homeostasis, cecal commensals (e.g., Ruminococcaceae, Lactobacillaceae) ferment dietary fiber to produce high concentrations of butyrate and acetate. These small metabolites cross the intestinal barrier into the circulation, acting as signaling metabolites that reach the lungs. This effectively establishes a gut-lung metabolite relay, allowing distal lung epithelial cells to utilize gut-derived metabolites (e.g., butyrate) to meet the energy demands of antiviral defense. Research confirms that circulating butyrate acts as a histone deacetylase (HDAC) inhibitor, upregulating the transcription of interferon-stimulated genes (ISGs) like *OASL* in respiratory epithelial cells via epigenetic modification, significantly enhancing innate immune defense against viruses like H9N2 avian influenza (Oladokun, 2025; Li, 2022). This process involves not only epigenetic modification but also precise molecular relay: gut-derived butyrate, as a key signaling molecule, can specifically enhance the binding activity of transcription factor Sp1 to the *OASL* promoter region, thereby engaging a post-transcriptional inhibition (i.e., the inhibition of viral mRNA translation)at the early stages of viral invasion. Furthermore, novel metabolites like pyrroloquinoline quinone (PQQ) have demonstrated potent antioxidant and immune-activating capacities, potentially synergizing with SCFAs to optimize the respiratory microenvironment. This metabolic axis constitutes the material basis for the gut-lung axis-mediated modulation of respiratory immunity by gut microbiota (Fig. 1).

### Homing and migration within the common mucosal immune system

Beyond molecular signals, the trans-organ migration of immune cells themselves provides a cellular link between gut and lung, a process dependent on the Common Mucosal Immune System (CMIS). Gut-associated lymphoid tissue (GALT, e.g., cecal tonsils) serves as a primary antigen-sensitization site. In mammalian models, gut-activated B and T cells upregulate specific homing receptors such as CCR9, CCR10, or CCR6, and migrate via lymph and blood to distant mucosal effector sites (Ni, 2023; Lin, 2020).Whether equivalent receptor-ligand pairs direct lymphocyte trafficking to the avian respiratory mucosa requires further investigation. Studies show that when respiratory mucosa highly expresses the chemokine CCL20, it can precisely recruit antigen-specific lymphocytes expressing CCR6, enabling the ectopic secretion of sIgA and systemic sharing of immune memory (Ni, 2023; Lin, 2020). This cellular migration mechanism ensures that antigens encountered in the gut can prime the respiratory immune system, moving the defensive front line forward.

### Virus-induced microecological vicious cycle

Gut-lung communication is markedly bidirectional and can form a vicious cycle under pathological conditions. Respiratory viral infections (e.g., AIV, IBV) not only cause local damage but can also induce intestinal dysbiosis via the release of inflammatory factors or neuroendocrine modulation (e.g., elevated corticosterone). This dysbiosis is characterized by decreased Firmicutes abundance and overgrowth of conditional pathogens like *E. coli* (Ngunjiri, 2021; Wang, 2024). The disrupted gut microenvironment further compromises the intestinal barrier, leading to the translocation of gut-derived pathogens or their toxins (e.g., lipopolysaccharide LPS) into the circulation. These can secondarily colonize the already damaged lung, causing lethal virus-bacteria co-infections. For instance, in mammalian models, intestinal mycobiota dysbiosis has been associated with *E. coli* translocation to the lungs (Wang et al., 2024a). Whether this pathway operates to the same extent in poultry requires direct investigation. This mechanism reveals that preventing the secondary collapse of gut microecology is a key strategy for halting the progression of avian respiratory viral infection into lethal systemic disease.

## Shaping of respiratory TRM cells by microenvironmental signals

If gut-derived metabolites provide the metabolic substrates for respiratory defense, then TRM are the frontline sentinel cells stably retained at the respiratory mucosa by these signals. In the avian respiratory tract, which lacks draining lymph nodes, the long-term local residency of memory T cells is a prerequisite for immediate defense—a process entirely dependent on the imprinting control by microenvironmental signals (Raha, 2025).

### TRM settling signals: microenvironmental imprinting and tissue retention

Since Erf (2004) laid the foundational characterization of avian cell-mediated immunity, TRM-cell differentiation and retention have been recognized as being regulated by both cell-intrinsic transcriptional programs and tissue-derived signals, with the latter playing a particularly prominent role in the respiratory mucosa. Transforming growth factor-beta (TGF-β), induced by epithelial cells or commensals, acts as a key retention signal. TGF-β signaling downregulates the expression of KLF2 and S1PR1 (which inhibit tissue egress) while upregulating the expression of CD103 (αEβ7 integrin) and CD69, promoting tight anchoring of T cells to E-cadherin on epithelial cells. Consistent with this mechanism, intranasal delivery of an adenovirus-vectored avian influenza vaccine in chickens has been shown to induce potent mucosal immune responses and protection against respiratory challenge, supporting the paradigm that the intranasal route effectively engages NALT antigen presentation and TGF-β-driven tissue retention pathways to establish local immunity (Nie et al., 2026).

### Metabolic reprogramming and functional maintenance: epigenetic regulation by gut-derived SCFAs

Given the above mechanisms, how do TRM cells maintain survival for months in the nutrient-limited, pathogen-rich respiratory mucosa? The core adaptive mechanism is metabolic reprogramming, and a key element in this process is the gut-lung axis metabolite discussed earlier: short-chain fatty acids (SCFAs). Mammalian studies show that microbiota depletion drastically reduces respiratory TRM cells, and SCFA supplementation can restore their abundance (Saint-Martin, 2024; Li, 2022b). Evidence from mammalian studies also suggests that SCFAs can influence T-cell metabolism and epigenetic regulation, and that TRM cells depend on lipid uptake and fatty-acid metabolism for long-term persistence in some tissues. However, a causal pathway linking avian cecal SCFAs, histone H3K9 acetylation, FABP4/5 expression, and respiratory TRM-cell maintenance has not been demonstrated. We therefore propose this pathway as a testable hypothesis requiring direct investigation in avian models.

### Dynamic interplay: immune sculpting of microecological structure

The interaction between TRM cells and microbiota is significantly bidirectional: microbiota program TRM, and in turn, TRM shape the microecology. TRM cells colonizing NALT and lungs function as ecological modulators by continuously secreting low levels of IL-17A and IFN-γ. IL-17A upregulates β-defensins and mucins produced by respiratory epithelial cells. This antimicrobial peptide barrier may contribute to colonization resistance by limiting pathogen access to the epithelial surface (Meunier, 2025; Zhang, 2023). The taxon-specific consequences of TRM-derived cytokines on microbial community structure remain largely unknown in birds. Furthermore, TRM-helper B cell-derived sIgA can coat commensals, restricting them to the outer mucus layer to prevent excessive immune activation (Huang et al., 2023; Wang, 2023). The establishment of this IL-17/sIgA-microbiota feedback loop ensures dominant microbiota occupy the respiratory ecological niche, effectively preventing secondary infections due to vacant niches.

## From local microecological imbalance to systemic defense failure

### Collapse of core microbiota homeostasis

The collapse of the microecological defense system often begins with stochastic perturbations induced by environmental stress or human intervention. Poultry are highly stress-sensitive; heat or transport stress causes a sharp rise in blood corticosterone, which has been associated with altered mucosal glycosylation patterns and reduced abundance of dominant commensals like Lactobacillaceae (Mangan, 2024). Beyond thermal stress, airborne fine particulate matter (PM2.5) in broiler houses represents an additional environmental disruptor of respiratory microecology. Zhou et al. (2023) demonstrated that PM2.5 exposure altered pulmonary microbiome β-diversity and selectively promoted the overgrowth of Enterococcus cecorum, while antibiotic-mediated pulmonary microbiota depletion significantly attenuated PM2.5-induced lung inflammation and suppressed IL-1β, TNF-α, and IL-6 expression. This causal evidence indicates that PM2.5-induced inflammation is at least partially mediated through microbiota dysbiosis, with PM2.5 altering the bacterial growth environment to favor pathobiont expansion.

A more potent factor is broad-spectrum antibiotic use, which can disrupt microbial community composition and reduce colonization resistance, although the magnitude and anatomical distribution of these effects vary among studies (Figueroa, 2020). This instantaneous depletion of core microbiota disrupts colonization resistance, vacating ecological niches for explosive proliferation of conditional pathogens like *E. coli* and *Mycoplasma*, becoming the initiating trigger event of pathology.

### Barrier failure and inflammatory polarization

Once microecological imbalance occurs, it triggers a chain reaction via the metabolism-immune axis. Lacking SCFAs and tryptophan metabolites normally produced by commensals, epithelial cells downregulate tight junction proteins (Claudin-1/Occludin), increasing physical barrier permeability (Saint-Martin, 2024). Pathogens and their products (e.g., LPS) subsequently penetrate the compromised mucosa and are recognized by TLR4 on lamina propria macrophages. In the absence of microecological tolerogenic signals, macrophages rapidly undergo metabolic reprogramming and adopt a pro-inflammatory activation state characterized by increased production of IL-1β, IL-6, and TNF-α (Wang et al., 2024a; Peng, 2023). This uncontrolled cytokine storm fails to clear pathogens and instead exacerbates epithelial apoptosis and vascular exudation, forming a vicious positive feedback cycle of inflammation-damage.

### The lethal pathway of breaching the blood-gas barrier

The comprehensive failure of local defense ultimately leads to systemic pathogen dissemination. Once the microecological shield is shattered, the avian blood-gas barrier, normally a site for gas exchange, is exposed and instantly becomes a direct conduit for pathogens to access systemic organs. When pulmonary inflammation damages epithelial-endothelial barrier integrity, pathogens (e.g., avian pathogenic *E. coli* (APEC) or highly pathogenic avian influenza virus) readily breach this fragile line into the circulation (Hu, 2025; Wu, 2022). The absence of conventional encapsulated lymph nodes in chickens and turkeys may modify the kinetics of pathogen clearance and immune organization following systemic entry; however, the direct contribution of this anatomical feature to hematogenous pathogen amplification has not been formally established. Once in circulation, pathogens can spread via the circulatory system to invade parenchymal organs such as the liver and kidneys, leading to bacteremia, viremia, and multi-organ failure (MOF). Additionally, intestinal mycobiota dysbiosis has been linked to E. coli lung translocation in mammalian models (Wang et al., 2024b), although the relevance of this mechanism to avian species remains to be established. Therefore, the progression from local microecological imbalance to systemic lethality is essentially the result of the sequential failure of the microecological, immune, and anatomical barriers.

## Constructing the host-microbiota-environment three-dimensional regulation model

### Tripartite interaction of trigger, mediator, and effector

**Fig. 2:**
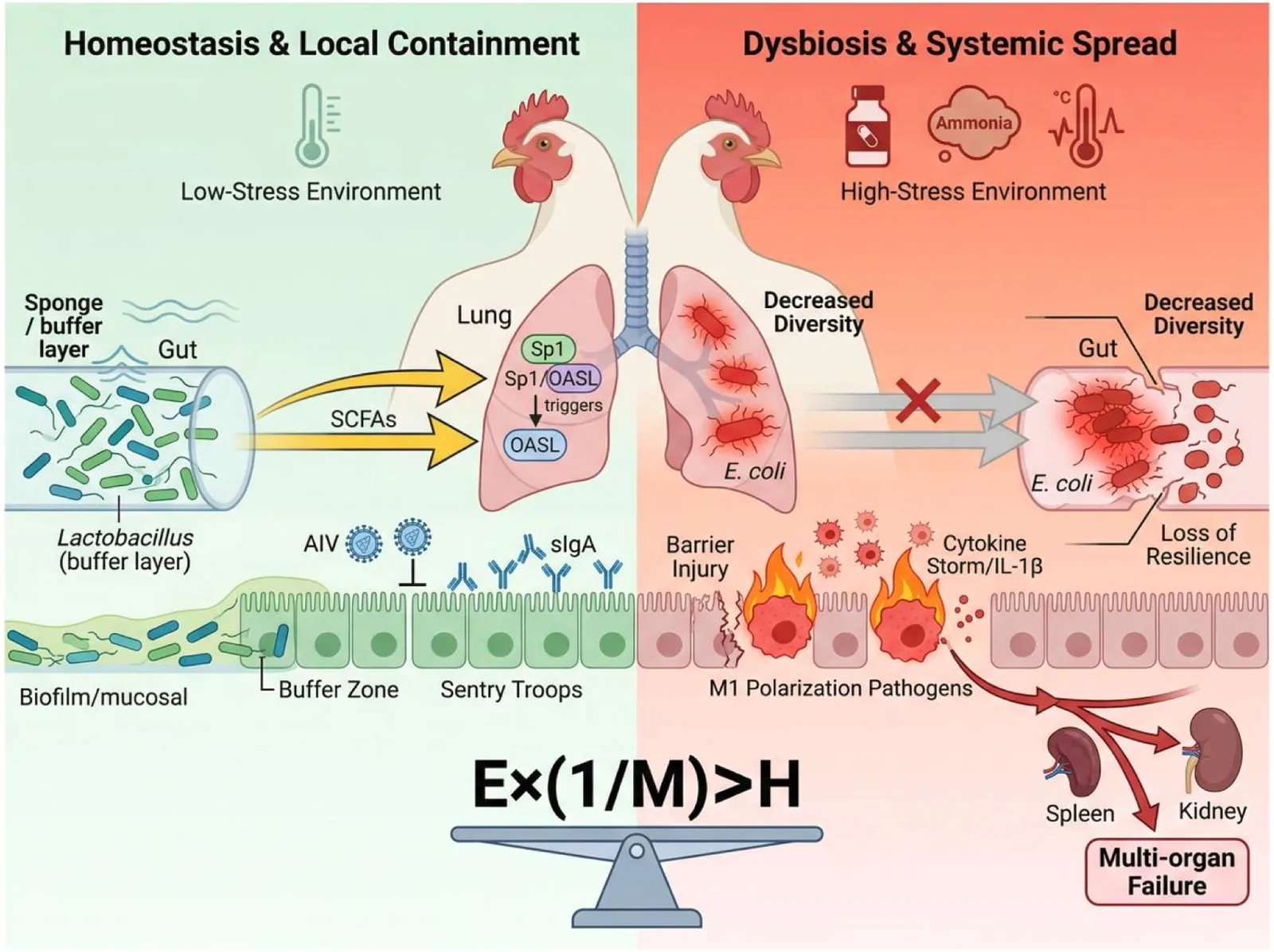
The "Three-Dimensional Regulation Model": From Local Homeostasis to Systemic Collapse. Description: This model illustrates the non-linear interaction between environmental stressors, microbial buffers, and host immunity. Left (Homeostasis & Defense): Under stable conditions, gut-derived SCFAs promote lung epithelial defense via the Sp1/OASL pathway and support the maintenance of tissue-resident memory T cells (TRM), containing pathogens locally. Right (Dysbiosis & Systemic Spread): Environmental stressors (e.g., antibiotics, ammonia, heat stress) disrupt the microbial buffer (Mediator), causing a threshold breach where E×(1/M) > H. This leads to a loss of "colonization resistance," triggering pro-inflammatory cytokine storms (e.g., IL-1β) and allowing pathogens to breach the blood-gas barrier for systemic dissemination to the spleen and kidneys.

Based on the preceding molecular mechanism analysis, we construct a three-dimensional "Environment (E)-Microbiota (M)-Host (H)" regulation model for avian respiratory health . This model visually illustrates the dynamic switch of the respiratory ecosystem between homeostatic defense and systemic collapse, moving beyond traditional single-cause theories to propose that disease emergence results from non-linear interactions. The environmental dimension includes trigger variables such as ammonia concentration, temperature fluctuations, and antibiotic use, which act as perturbation inputs, setting the baseline pressure on the immune system. The microbial dimension features mediator variables like dominant microbiota (e.g., Lactobacillaceae) and their metabolites (e.g., SCFAs), which act as buffers, regulating host immune thresholds through mechanisms like metabolic reprogramming (e.g., HDAC inhibition) and determining whether environmental pressure translates into pathological damage. The host immune dimension comprises effector variables like NALT, TRM cells, and the sIgA barrier—the ultimate executors responsible for pathogen clearance and tissue repair. The model's core hypothesis is that disease risk may increase when environmental pressures exceed the combined buffering capacities of the microbial community and host defense system, rather than being determined solely by pathogen presence. This relationship is proposed as a heuristic framework and has not been quantitatively parameterized or validated (Agarwal, 2024) (Fig. 2).

### Thresholds of homeostasis and disease: ecological resilience and the tipping point

This model explains the common clinical phenomenon of "variable disease susceptibility within a flock." We introduce the concept of ecological resiliencethe-the ability of a microbial community to recover its original structure after disturbance. Individuals with high resilience possess more complex and interconnected microbial networks, capable of absorbing environmental stress (e.g., corticosterone fluctuations from heat stress) like a buffering capacity, preventing direct impact on the immune system (Davis, 2023). However, the system possesses an ecological threshold. Once antibiotic abuse or sustained adverse environmental conditions drives microbial diversity below this tipping point, the microbial buffer function approaches zero, and even minor pathogenic stimuli can trigger a severe cytokine storm and systemic infection.Therefore, the core of prevention and control should not merely be pathogen eradication but enhancing the system's ecological resilience through microecological interventions (e.g., prebiotic supplementation) to keep it away from the brink of collapse.

### Model generalizability and species-specific variations

This model is primarily constructed based on landfowl (Galliformes, e.g., chickens, turkeys), whose core feature is the lack of draining lymph nodes, leading to high dependence on NALT and local microecology for compensation. However, for waterfowl (Anseriformes, e.g., ducks, geese), the model requires specific modifications. Comparative immunology studies show that ducks and geese have evolved more developed and sophisticated lymphatic structures (e.g., cervicothoracic lymph node-like structures) and more robust innate immune receptors (e.g., a fully functional RIG-I pathway), granting them greater systemic tolerance to influenza viruses and often enabling them to act as asymptomatic reservoirs (Figueroa, 2020b; Feng, 2024). In contrast, chickens lack a functional RIG-I pathway and possess a more fragile blood-gas barrier, resulting in markedly lower tolerance for "microecology-immune" imbalances and a high propensity for lethal immunopathology. Therefore, when applying the "three-dimensional model" to guide disease control in chickens, greater emphasis should be placed on environmental management and maintaining microecological homeostasis. For waterfowl, the focus should center on their role as potential reservoirs and the associated risk of pathogen spillover to landfowl.

The framework generates three testable predictions: (1) identical environmental stressors will produce greater inflammatory responses in birds with lower microbial resilience; (2) restoration of microbial function will improve barrier-related outcomes without necessarily eliminating pathogen exposure; and (3) the relative contribution of host, microbial, and environmental components will differ between chickens and waterfowl. These predictions provide a roadmap for future experimental validation of the proposed framework.

## From target discovery to precision intervention: towards "ecological health farming"

### High-throughput technology-driven target discovery: decoding the microecology-immune interactome

Moving from descriptive to mechanistic research, multi-omics integration is the core strategy for identifying key targets. Metagenomics now focuses beyond species annotation to mining functional gene clusters—for instance, precisely identifying key genes encoding bacteriocins or tryptophan-metabolizing enzymes in *Lactobacillus salivarius* to clarify their molecular basis for maintaining colonization resistance. Concurrently, single-cell RNA sequencing can resolve mucosal immune cell heterogeneity with high resolution, enabling precise sorting of TRM cell subsets highly expressing CD69/CD103 and revealing their specific transcriptional signatures (e.g., upregulated *FABP* expression), providing direct evidence to validate the metabolic reprogramming hypothesis (Liu, 2021; Liukang, 2024). This cross-boundary correlative analysis of microbial functional profiles and host cellular profiles is a prerequisite for discovering novel intervention targets.

### Next-generation microecological intervention strategies: the rise of postbiotics and engineered bacteria

Traditional probiotic interventions are often limited by low colonization efficiency and environmental instability. Therefore, we propose a strategic shift towards postbiotics and synthetic biology. The postbiotic substitution strategy involves the direct delivery of well-defined, active microbial metabolites (e.g., specific SCFAs, PQQ, or defined bacterial lysates). This approach circumvents the variability of live bacteria colonization, allowing for rapid, dose-dependent activation of epithelial barrier genes (e.g., OASL) or inhibition of inflammatory pathways, offering greater biosafety and stability (Saint-Martin, 2024; Kusampudi, 2025). The engineered bacteria strategy utilizes synthetic biology to modify chassis strains such as engineered Lactococcus to deliver defined immunomodulatory molecules (e.g., sIgA, antimicrobial peptides, or IL-22) at mucosal surfaces (Awaad, 2023; Hewawaduge, 2023). At present, this strategy remains largely experimental, and its stability, containment, biosafety, and regulatory feasibility in poultry production require evaluation. This strategy enables dynamic, precise repair of microecological imbalance, representing a frontier direction for future ecological intervention therapy.

### Gene editing and novel vaccines: building durable immune barriers

**Fig. 3:**
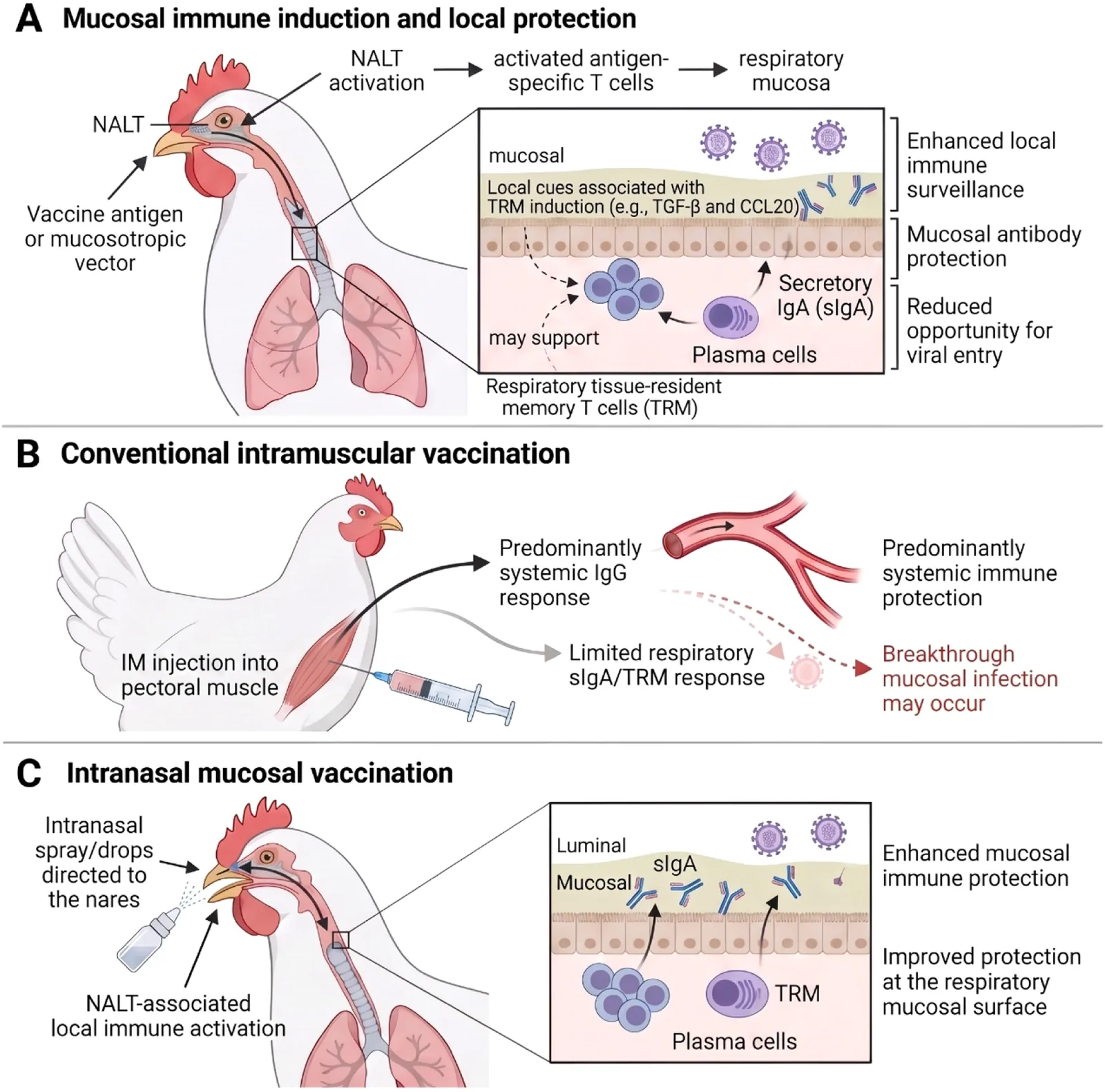
Conceptual comparison of conventional intramuscular and intranasal mucosal vaccination strategies for respiratory immune protection in poultry. (A) Vaccine antigen exposure at the nasal mucosa may activate local immune responses in nasal-associated lymphoid tissue (NALT) and support the establishment of respiratory tissue-resident memory T cells (TRM) and mucosal antibody responses. (B) Conventional intramuscular vaccination is administered into the pectoral muscle and primarily promotes systemic immune responses, whereas protection at the respiratory mucosal surface may be limited. (C) Intranasal vaccination delivers vaccine material to the nares and is designed to enhance local mucosal immunity, including secretory immunoglobulin A (sIgA) and respiratory TRM-associated protection. The scheme is conceptual and does not imply that all pathways have been demonstrated directly in avian species.

Beyond microecological regulation, enhancing host resistance is another defensive path. CRISPR/Cas9 gene editing is already used for genome-wide screening to identify and knockout host factors such as ANP32A that influenza virus replication depends on and may support the development of genetically informed resistance strategies (Li, 2025; Zhou, 2025). Translation to commercial poultry breeding will require evaluation of efficacy, off-target effects, animal welfare, heritability, and regulatory acceptability. In vaccine development, novel mucosal vaccines based on TRM induction mechanisms represent a breakthrough. Using mucosotropic vectors like adenovirus or attenuated *Salmonella* to deliver pathogen antigens while co-expressing TRM-inducing molecules like TGF-β or CCL20 can efficiently activate NALT and promote the homing and residency of antigen-specific T cells to the lungs, establishing a durable frontline immune barrier (Aganja, 2025). By mimicking the signaling cascade of natural infection, next-generation mucosal vaccines, compared to traditional routes, can significantly enhance memory T cell residency, building a more robust defensive barrier against re-infection. This combined integrated genetic and microecological approach strategy aims to construct a comprehensive, multi-layered defense network (Fig. 3).

### Challenges and future perspectives: crossing the valley of death in translational medicine

Despite promising prospects, this field faces three major translational barriers.Data standardization is the primary challenge; current microbiome data lack unified quality control and metadata standards, severely limiting cross-study comparisons and biomarker discovery(Wang, 2023b; Kursa, 2022). causality verification constitutes the core scientific bottleneck, and most studies merely stay at the descriptive stage where microbiota changes are only correlated with disease. Future work must utilize germ-free avian models and fecal microbiota transplantation (FMT) techniques to rigorously establish causal chains linking "specific bacterial strains/metabolites → immune phenotypes" (Kusampudi, 2025; Gaulke, 2025). The Clinical Translation Gap is a practical hurdle; vast differences exist between laboratory settings and intensive farming environments (e.g., ammonia, dust, stress). The stability of microecological preparations and the cost-effectiveness of delivery systems (e.g., nano-encapsulation) are critical determinants for large-scale application (Serian, 2023; White, 2022).

## Conclusion

This review synthesizes recent advances in immunology, microbiome research, and comparative biology to systematically elucidate the defensive logic of the avian respiratory tract as an open ecosystem. Current evidence indicates that the respiratory microbiota is not a passive microbial reservoir but functions as an integral component of the mucosal immune system. Dominant commensals, represented by *Lactobacillaceae*, not only maintain physical barrier integrity via metabolites (SCFAs, PQQ) but are also likely involved in the epigenetic imprinting, differentiation, and functional maintenance of TRM, thereby compensating for the anatomical void left by the absence of draining lymph nodes.

Based on this, we propose a "Host Immunity-Microbiota-Environmental Factors" three-dimensional regulation model. This model not only clarifies the non-linear mechanism of disease emergence,where local microecological imbalance, triggered when environmental stressors exceed the combined buffering capacity of the microbiota and the host immune threshold, escalates through cascade amplification into lethal systemic infection, but also provides a theoretical explanation for current challenges in the poultry industry, such as variable disease susceptibility within flocks and insufficient vaccine protection.

Looking ahead, the prevention and control of avian respiratory diseases may benefit from integrating microbiota-directed interventions with vaccination, biosecurity, environmental management, and responsible antimicrobial use, emphasizing microbiota-immune synergy and ecological resilience as complementary principles rather than replacements for existing measures. By applying next-generation microecological interventions like postbiotics and engineered bacteria to consolidate system homeostasis, combined with novel mucosal vaccines designed to target and induce TRM, we can potentially construct more robust endogenous biological defenses. This approach may ultimately contribute to healthier and more sustainable poultry production.

## Ethics approval and consent to participate

Not applicable.

## Consent for publication

Not applicable.

## Availability of data and materials

Not applicable.

## Competing interests

Qingrong Jiang, Xin Zhao, and Miaoxi Deng are employees of Sichuan Sundaily Ecological Food Co., Ltd. The remaining authors declare that they have no competing interests.

## Declaration of generative AI and AI-assisted technologies in the manuscript preparation process

During the preparation of this work the authors used ChatGPT, Doubao, Grammarly, DeepL and jova.ai in order to improve language expression, polish the manuscript, check grammar, assist in manuscript organization, and prepare scientific figures. After using these tools/services, the authors reviewed and edited the content as needed and takes full responsibility for the content of the published article.

## Funding

The authors received no specific funding for this work.

## Author Contribution

**Miao Yin:** Conceived the study, designed the research framework and experimental protocols, performed the experiments and formal data analysis, curated and managed the research data, developed relevant software programs, provided experimental resources, supervised the whole research work, administered the research project, validated the experimental results, created the figures and visualizations, and wrote the original manuscript. **Xuefeng Qi:** Designed experimental approaches, acquired financial support for this research, and critically revised the manuscript. **Qingrong Jiang:** Developed relevant software programs, designed experimental approaches, and curated and managed the research data. **Lizhen Wang:** Designed experimental approaches, developed relevant software programs, provided experimental resources, curated and managed the research data, validated the experimental results, created figures and visualizations, supervised part of this study, and wrote the original manuscript. **Qian Wu:** Designed experimental approaches, performed experiments and formal data analysis, curated and managed the research data, developed relevant software programs, provided experimental resources, administered the research project, and created figures and visualizations. **Yu Xia:** Designed experimental approaches, developed relevant software programs, provided experimental resources, validated the experimental results, supervised part of this study, and created figures and visualizations. **Xin Zhao:** Designed experimental approaches, provided experimental resources, performed formal data analysis, and curated and managed the research data. **Miaoxi Deng:** Developed relevant software programs, provided experimental resources, administered the research project, performed formal data analysis, and curated and managed the research data. **Xuemei Ding:** Designed experimental approaches, validated the experimental results, supervised part of this study, and created figures and visualizations. **Jinglong Yang:** Designed experimental approaches, performed experiments and formal data analysis. **Xiwen Chen:** Designed experimental approaches and acquired financial support for this research.

## Disclosures

The authors declare that they have no known competing financial interests or personal relationships that could have appeared to influence the work reported in this paper.
